# How Do Personal Attributes Shape AI Dependency in Chinese Higher Education Context? Insights from Needs Frustration Perspective

**DOI:** 10.1371/journal.pone.0313314

**Published:** 2024-11-01

**Authors:** Wenjun Zhong, Jianghua Luo, Ya Lyu

**Affiliations:** 1 Center for Studies of Education and Psychology of Ethnic Minorities in Southwest China, Southwest University, Chongqing, China; 2 College of Educational Science, Xinjiang Normal University, Urumqi, Xinjiang, China; Instituto Superior de Contabilidade e Administracao de Lisboa - Instituto Politecnico de Lisboa (ISCAL-IPL), PORTUGAL

## Abstract

**Objective:**

The adoption of Generative AI in education presents both opportunities and challenges, particularly regarding its potential to foster student dependency. However, the psychological drivers of this dependency remain unclear. This study addresses this gap by applying the Interaction of Person-Affect-Cognition-Execution (I-PACE) model and Basic Psychological Needs (BPN) theory to explore how specific personality traits—neuroticism, self-critical perfectionism, and impulsivity—contribute to AI dependency through needs frustration, negative academic emotions, and reinforced performance beliefs.

**Method:**

Data were collected from 958 university students (*M*_age_ = 21.67) across various disciplines. Structural equation modeling (SEM) was used to analyze the relationships among the variables.

**Results:**

Neuroticism, self-critical perfectionism, and impulsivity were found to be significantly associated with increase needs frustration and negative academic emotions, which in turn reinforced students’ positive beliefs about performance of AI tools, deepening their dependency. The study also uncovered complex serial mediation effects, highlighting intricate psychological pathways that drive maladaptive AI use.

**Conclusions:**

This research provides a critical insight into the interplay between personality traits and technology use, shedding light on the nuanced ways in which individual differences influence dependency on generative AI. The findings offer practical strategies for educators to promote balanced AI use and support student well-being in educational settings.

## Introduction

The rapid integration of digital technologies into daily life has significantly influenced how individuals, particularly students, interact with information and educational tools. This transformation has garnered the attention of personality psychologists, who are increasingly focused on understanding the role of individual differences in shaping these interactions [1, 2]. A notable development in this digital landscape is the emergence of Generative AI tools like ChatGPT, which have revolutionized the way students and professionals engage with educational and professional contents [3].

While Generative AI tools offer advanced capabilities, such as personalized learning experiences, immediate feedback, and the potential to enhance productivity [4, 5]. Their rapid adoption has also raised concerns, particularly regarding over-reliance in educational contexts [6]. This dependency raised concerns about academic integrity problem, as compromising academic integrity can significantly impair the reputation of higher education institutions and diminish students’ intrinsic motivation [7]. Moreover, reliance on AI can impede the development of critical thinking skills, as students may become accustomed to seeking quick answers rather than engaging in deeper analytical processes [8, 9]. The conveniences offered by these tools can also foster procrastination, as students may defer tasks by relying on AI to provide last-minute assistance [10].

Despite the growing use of ChatGPT in educational settings, most research has focused on the technical attributes and educational benefits of these tools, while overlooking the psychological mechanisms that drive dependency [11]. This technology-centric approach neglects critical user-centered factors—particularly personal attributes—that could contribute to maladaptive technology use. Although recent studies have begun to explore contextual influences such as workload, time constraints, as well as stress level [10, 12], the role of individual psychological states and personality traits in fostering dependency remains underexplored. Addressing this gap is crucial for understanding the broader implications of generative AI in education and ensuring its responsible use [13].

Traits such as neuroticism, perfectionism, impulsivity, and low self-esteem have been consistently linked to maladaptive behaviors in digital contexts, including excessive social media use and internet addiction [14–16]. Individuals with these traits often experience psychological distress, which can intensify in high-pressure academic environments. To investigate how these personality traits influence the development of dependency on ChatGPT, this study applies the Interaction of Person-Affect-Cognition-Execution (I-PACE) model and Basic Psychological Needs (BPN) theory. Specifically, the study aims to answer the research question: “How does the interplay between personality traits and cognitive-emotional experiences shape the tendency to develop dependency on ChatGPT in educational environments?” By exploring the interaction between personality traits and cognitive-emotional experiences, this research aims to fill the existing gap in the literature on AI dependency in educational settings. The findings are expected to contribute to the theoretical discourse in personality psychology and provide practical insights for educators and technology developers. More broadly, our research offers new perspectives on the dynamics of human‒machine interactions in the era of Generative AI.

## Theoretical framework and hypothesis formulation

### I-PACE framework

The Interaction of Person-Affect-Cognition-Execution (I-PACE) model, proposed by Brand [17], offers a comprehensive framework for understanding the development of maladaptive behaviors in digital contexts. This model suggests that the interplay between personal characteristics (e.g., personality traits), affective responses, cognitive processes, and executive functions drives such behaviors. In this study, we focus on personality traits as key personal characteristics that influence how individuals experience and respond to their environment. Specifically, needs frustration, shaped by these personality traits, acts as a subjective experience that triggers affective and cognitive responses. Cognitive responses, such as performance expectations of ChatGPT, play a crucial role in this process. These expectations represent biases about the tool’s effectiveness in enhancing academic performance. When students develop high performance expectations, they may become overly reliant on ChatGPT, believing that it can help them achieve better results with minimal effort [18, 19]. This excessive reliance can lead to maladaptive patterns of technology use, consistent with the pathways described by the I-PACE model.

### Needs frustration and ChatGPT dependency

According to Basic Psychological Needs (BPN) theory, the frustration of needs for autonomy, relatedness, and competence can lead to negative psychological outcomes, such as increased stress, reduced motivation, and reliance on external coping mechanisms [20]. Przybylski [21] found that individuals with frustrated psychological needs tend to turn to video games as a source of competence and relatedness, suggesting a similar pattern may apply to other forms of technology. Research by Kardefelt [22] indicated that individuals with higher levels of needs frustration are more likely to develop problematic behaviors with technology use since internet addiction can be considered compensatory to alleviating discomfort raised from state of frustration. In line with previous research findings, ChatGPT can serve as a coping mechanism for unmet psychological needs by providing immediate responses and simulated interactions, thereby fulfilling students’ needs for competence and autonomy [23, 24]. Thus, we hypothesize that:

**H1**: Needs frustration is positively associated with ChatGPT dependency.

### Personality as an antecedent

Personality traits significantly influence how individuals perceive and respond to their environments, particularly in academic settings. Traits such as neuroticism, self-critical perfectionism, and impulsivity predispose individuals to heightened needs frustration and negative academic emotions, which can lead to increased dependency on ChatGPT.

Neuroticism is characterized by a tendency to experience negative emotions, such as anxiety, depression, and irritability [25]. Individuals with high neuroticism are more sensitive to stressors and often perceive situations as threatening, leading to frustration with unmet psychological needs, particularly autonomy, relatedness, and competence [26]. This sensitivity can exacerbate emotions like anxiety, fear, and guilt, further contributing to negative academic experiences [27].

Self-Critical Perfectionism involves setting excessively high personal standards and being overly critical of one’s performance [28]. This trait is associated with chronic self-doubt and fear of failure, resulting in a persistent sense of inadequacy and unmet needs for competence and autonomy [29]. Such individuals are prone to anxiety, depression, and guilt, particularly in academic contexts where they often perceive themselves as falling short of their high expectations [30].

Impulsivity is defined by a tendency to act without forethought, often leading to difficulties in self-control and decision-making [31]. Impulsive individuals may struggle to meet their basic psychological needs due to their inability to plan or sustain effortful activities, leading to frustration [32]. This trait is also linked to increased feelings of frustration, anger, and regret, especially when academic setbacks occur as a result of poor planning [33].

**H2:** Personality traits (neuroticism, self-critical perfectionism, and impulsivity) are positively associated with needs frustration.
**H3:** Personality traits (neuroticism, self-critical perfectionism, and impulsivity) are positively associated with negative academic emotions.

### Negative academic emotions and performance expectations

Negative academic emotions, such as anxiety and hopelessness, are common in educational settings and have been shown to impair attention and academic performance [34, 35]. These emotions are often a direct consequence of needs frustration [36] and may drive students to seek external coping mechanisms like ChatGPT to manage their stress. Additionally, performance expectations refer to the extent to which individuals believe that using technology enhances their performance. It is recognized as a crucial cognitive factor influencing attitudes and behavioral intentions to use technology, as justified by the Unified Theory of Acceptance and Use of Technology (UTAUT) model [37]. The I-PACE model proposes that cognitive bias and illusion, the belief that technology can provide efficient and accurate answers, can bridge personal and psychological factors and problematic technological behavior. Therefore, we hypothesize that:

**H4:** Needs frustration positively influences negative academic emotions.
**H5:** Negative academic emotions are positively associated with ChatGPT dependency.
**H6:** Needs frustration is positively associated with performance expectations.
**H7:** Negative academic emotions are positively associated with performance expectations.
**H8:** Performance expectations are positively associated with ChatGPT dependency.

### Serial mediation effects

**Fig 1:**
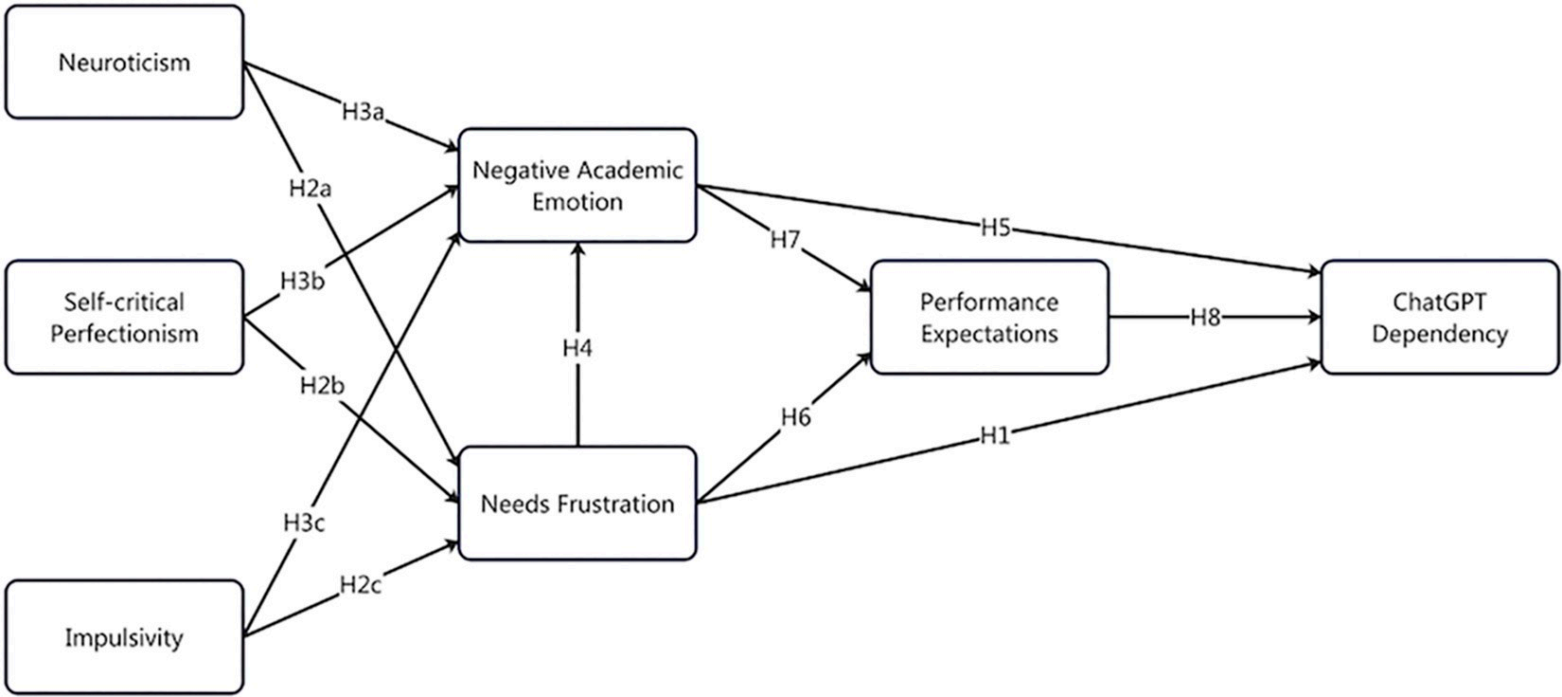
Proposed research model.

To understand the comprehensive relationship between personality traits, needs frustration, negative academic emotions, performance expectations, and ChatGPT dependency, we propose a serial mediation model (Fig 1). This model hypothesizes that personality traits (neuroticism, self-critical perfectionism, and impulsivity) indirectly influence ChatGPT dependency through a sequence of mediators—needs frustration, negative academic emotions, and performance expectations. Specifically, we posit that the three proposed personality traits, including neuroticism, self-critical perfectionism, and impulsivity, predict higher levels of needs frustration. This subjective psychological state increases negative academic emotion and performance expectations, consequently leading to ChatGPT dependency.

## Materials and methods

### Participants and procedure

Participants were recruited online through a reputable survey platform named “Sojump,” a prominent platform in China specializing in online questionnaires. We obtained ethical approval from IRB of Xinjiang Normal University (No. 1076220240523003). The recruitment started on 5^th^ June 2024 and ended on 12^th^ June 2024. Upon expressing interest in the study, the participants were provided with a written informed consent form outlining the study’s purpose, procedures, potential risks, and benefits. All participants provided written informed consent before participation. The participants were assured of the confidentiality and anonymity of their responses. Full-time undergraduate and postgraduate students with experience using ChatGPT for academic work were targeted. To ensure data quality, duplicate submissions (identified by IP address) were excluded. Of the 1,000 students invited, 958 provided valid responses. Participants ranged from 18 to 28 years old (*M* = 21.67, *SD* = 2.46). The sample was representative of the broader student population in terms of gender and academic background (Table 1).

**Table 1:** Demographic statistics of participants (*N* = 958).

Measures	Item	Number	Percent
Gender	Male	494	51.6%
	Female	464	48.4%
Degree	Bachelor	675	70.5%
	Master	186	19.4%
	Ph.D.	97	10.1%
Study discipline	Social science/Humanities	174	18.2%
	Natural science	279	29.1%
	Engineering	212	22.1%
	Medicine	183	19.1%
	Art and Sports	110	11.5%
ChatGPT use frequency	Everyday	404	42.2%
	Always	389	40.6%
	Occasionally	104	10.8%
	Seldom	61	6.4%

### Measurements

In addition to demographic information, including gender, age, study discipline, degree level, and frequency of ChatGPT use, the questionnaire encompassed all constructs outlined in the proposed research model. The items were derived from instruments that demonstrated robust reliability and validity in prior studies. Minor modifications were made to some items to better fit our research aim, and these modifications were agreed upon by the three researchers involved in this study. To ensure measurement precision within the Chinese context, one researcher translated the original English instruments into Chinese, followed by back translation by another bilingual researcher.

**Neuroticism** was assessed using the 12-item subscale of the NEO Five-Factor Inventory (NEO-FFI) [38]. The neuroticism subscale comprises 12 items that evaluate participants’ proneness to psychological distress and emotional instability. Participants responded to each item on a 5-point Likert scale ranging from 1 (strongly disagree) to 5 (strongly agree). The scale exhibits strong reliability (McDonald’s ω = .94).

**Impulsivity** was measured by the Barratt Impulsiveness Scale-Short Form (BIS-15) [39]. The scale consists of 15 items (McDonald’s ω = .967) evaluating three factors of impulsivity: Non-planning impulsivity (McDonald’s ω = .906), which measures tendency to act without planning; motor impulsivity (McDonald’s ω = .908), which reflects spontaneous and unpremeditated action; and attentional impulsivity (McDonald’s ω = .905), which measures the inability to focus attention and tendency to make careless decisions. The responses were recorded on a 4-point Likert scale ranging from 1 (rarely/never) to 4 (almost always) according to the original version of the scale. To simplify the structural model while retaining the integrity of the measurement, the scores for each of the three impulsivity subfactors were averaged and rescaled to a 5-point scale. This process created composite scores for each sub-factor, which were used as indicators in the structural model.

**Self-critical perfectionism** was assessed via the 6-item subscale of the Big Three Perfectionism Scale-Short Form (BTPS-SF) [40], evaluating individuals’ concern about mistakes and high self-imposed standards. All items are graded on a 5-point Likert scale, ranging from 1 (strongly disagree) to 5 (strongly agree). In this study, the scale of self-critical perfectionism demonstrated acceptable reliability (McDonald’s ω = .876).

**Needs frustration** was assessed via the Basic Psychological Needs Satisfaction and Frustration Scale (BPNSFS) [41]. The scale measures the degree to which individuals feel that their psychological needs are unmet, aligning with the self-determination theory. The needs frustration scale comprises 12 items (McDonald’s ω = .942), measuring autonomy frustration (McDonald’s ω = .846), relatedness frustration (McDonald’s ω = .847), and competence frustration (McDonald’s ω = .838). Each factor was assessed using four items on a 5-point Likert scale ranging from 1 (not all true) to 5 (very true). Composite scores for autonomy, relatedness, and competence frustration were computed and used as indicators in the structural model.

**Negative academic emotion** was measured using the modified 12-item Achievement Emotions Questionnaire-Short Form (AEQ-S) [42], covering irritation, anxiety, and hopelessness. It demonstrated good reliability (McDonald’s ω = .94). All items were rated on a 5-point Likert scale ranging from 1 (strongly disagree) to 5 (strongly agree). We averaged the item scores based on the factor structure to form composite scores for irritation, anxiety, and hopelessness in the structural model.

**The performance expectation of ChatGPT** was evaluated with a modified 6-item scale based on perceived service quality from mobile providers [43]. The Revised scale specifically targets students’ perceptions of ChatGPT performance in supporting their academic activities (McDonald’s ω = 0.895). It consists of six items measured on a 5-point Likert scale ranging from 1 (strongly disagree) to 5 (strongly agree). Sample statements include “The information provided by ChatGPT is accurate and reliable for my academic work,” and “ChatGPT provides me with prompt assistance for my study-related queries.”

**ChatGPT dependency** was measured with 6 items adapted from prior studies [12, 44]. Sample items included “I tried to reduce my use of ChatGPT to help with my assignment, but I failed,” and “I feel that I relied on using ChatGPT to help complete my academic task more and more.” Each item is measured on a 5-point Likert scale ranging from 1 (strongly disagree) to 5 (strongly agree). The reliability index indicated acceptable internal consistency (McDonald’s ω = 0.863).

### Statistical analysis

Data analysis was conducted using SPSS and AMOS to rigorously evaluate the hypothesized relationships within the theoretical framework. The analysis commenced with comprehensive descriptive statistics, including means, standard deviations, and zero-order correlations of all study variables. To assess the potential common method bias, Harman’s single-factor test was performed by conducting an exploratory factor analysis (EFA) on all scale items without rotation to determine the amount of variance explained by a single factor. An acceptable result is that no single factor accounts for more than 50% of the total variance [45]. Additionally, the unmeasured common latent method factor technique was integrated into our confirmatory factor analysis (CFA), allowing each item to load onto both its theoretical construct and common latent method factor, further mitigating method-induced variance [46].

Construct validity was supported by factor loadings above 0.70, Average Variance Extracted (AVE ≥ 0.50), and Composite Reliability (CR ≥ 0.70) [47]. Discriminant validity was confirmed using the Heterotrait-Monotrait ratio (HTMT < 0.85) [48]. The overall structural model fit was assessed using several fit indices with the following standards: Values for the Comparative Fit Index (CFI) of ≥ 0.95, Goodness of Fit Index (GFI) of ≥ 0.90, Root Mean Square Error of Approximation (RMSEA) of ≤ 0.06 and Standardized Root Mean Square Residual (SRMR) of ≤ 0.08 indicate a good fit [49]. Path coefficients were considered significant at *p* < 0.05. Mediation analysis was conducted to evaluate the indirect effects. Robustness tests, including bias-corrected bootstrapping with 5,000 iterations for indirect effects, were performed to ensure the stability and reliability of the findings [50].

## Results

### Measurement model

#### Descriptive statistics and common method bias

S1 Table shows the Pearson correlation coefficients between all constructs. All correlations were significant (*p* < .01). Harman’s single-factor test indicated that common method bias was not a concern (variance = 23.902%). Additionally, the comparison with the CLF technique revealed that the addition of the common latent factor did not significantly improve the model fit (Δχ^2^/Δ*df* = 43.27/1, *p* > .05) confirming absence of common method bias.

#### Test of measurement validity

Table 2 presents the results of the fit indices for all the constructs in the unidimensional item-level CFA model. These results confirm that the constructs, both first- and second-order, are well represented by their respective items. Confirmatory Factor Analysis supported the validity of the constructs, with all factors loading greater than .70 (S2 Table), all CR value were greater than .70, and AVE were greater than .50 (Table 3). Discriminant validity was confirmed with all HTMT ratio was less than .85 (Table 4).

**Table 2:** Fit indices for item-level CFA.

Constructs	χ^2^/*df*	CFI	GFI	TLI	RMSEA	SRMR
Neuroticism	1.112	.999	.99	.999	.011[.00-.024]	.012
Self-Critical Perfectionism	1.007	1.00	.997	1.00	.003[.00-.037]	.01
Non-Planning Impulsivity	1.210	1.00	.997	.999	.015[.00-.049]	.007
Motor Impulsivity	1.05	1.00	.998	1.00	.007[.00-.046]	.006
Attentional Impulsivity	1.942	.998	.996	.997	.031[.00-.061]	.009
Impulsivity (2nd order)	1.126	.999	.987	.999	.011[.00-.022]	.01
Autonomy Frustration	.368	1.00	1.00	1.00	.00[.00-.048]	.004
Relatedness Frustration	4.958	.995	.995	.985	.064[.029-.107]	.014
Competence Frustration	.159	1.00	1.00	1.00	.00[.00-.048]	.003
Needs Frustration (2nd order)	1.435	.997	.988	.996	.021[.008-.032]	.013
Irritation	1.096	1.00	.999	1.00	.01[.00-.066]	.007
Anxiety	1.205	1.00	.999	.999	.015[.00-.068]	.007
Hopelessness	1.046	1.00	.999	1.00	.007[.00-.065	.006
Negative Academic Emotion (2nd order)	1.143	.999	.99	.999	.012[.00-.025]	.012
Performance Expectation	1.917	.997	.994	.995	.031[.005-.053]	.012
ChatGPT Dependency	.778	1.00	.998	1.00	.00[.00-.03]	.009

*Note*. Impulsivity, needs frustration, and negative academic emotion were modeled according to factor structures, with the general factor loaded on three subfactors, which were loaded by their constituent items. A 90% confidence interval (CI) is provided for RMSEA within brackets.

**Table 3:** CR and AVE of structural constructs.

Constructs	CR	AVE
Neuroticism	.94	.567
Self-Critical Perfectionism	.877	.542
Impulsivity	.967	.908
Needs Frustration	.942	.845
Negative Academic Emotion	.94	.838
Performance Expectation	.895	.586
ChatGPT Dependency	.863	.512

*Note*. The second-order constructs of impulsivity, needs frustration, and negative academic emotion were analyzed using composite scores derived from their respective first-order constructs. This approach was adopted to reduce complexity and keep model parsimony while maintaining theoretical rigor.

**Table 4:** Heterotrait-Monotrait (HTMT) ratio of structural constructs.

Constructs	1	2	3	4	5	6	7
**1** Neuroticism							
**2** Self-Critical Perfectionism	.224						
**3** Impulsivity	.218	.223					
**4** Needs Frustration	.24	.288	.241				
**5** Negative Academic Emotion	.32	.255	.222	.391			
**6** Performance Expectation	.316	.277	.26	.467	.48		
**7** ChatGPT Dependency	.281	.171	.191	.26	.204	.325	

### Structural model analysis

#### Test of model fit

The structural model demonstrated an acceptable fit (χ^2^/*df* = 1.139, CFI = .996, GFI = .96, TLI = .96, RMSEA = .012[.007-.016], SRMR = .047). Despite the data not exhibiting multivariate normality, maximum likelihood estimation was used. Bias-corrected bootstrapping with 5,000 resamples addressed potential non-normality biases, showing no significant change in standard errors (S3 Table).

#### Direct path analysis

**Fig 2:**
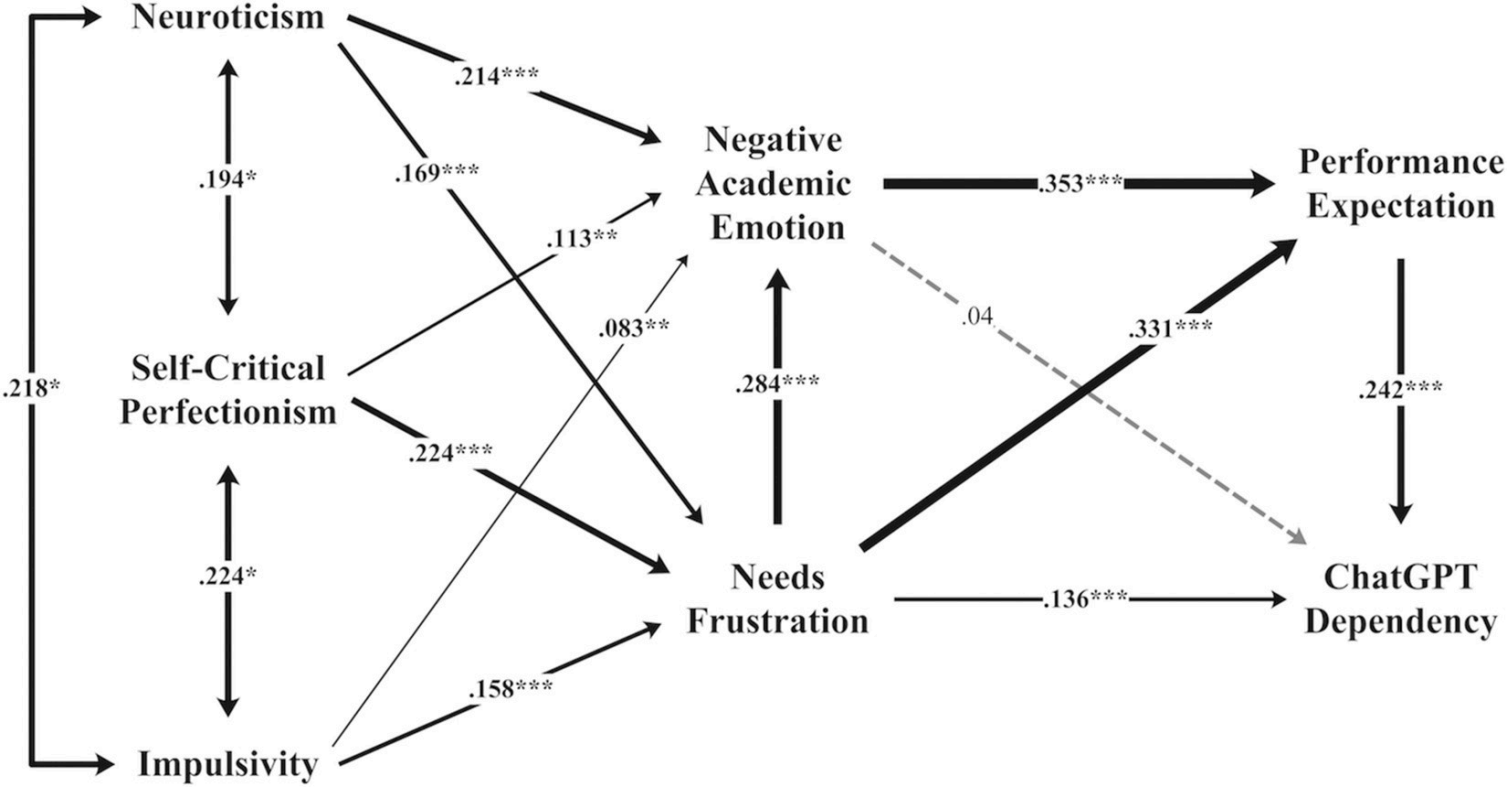
Structural model results.

SEM revealed significant relationships between personality traits and needs frustration, with neuroticism (β = .169), self-critical perfectionism (β = .224), and impulsivity (β = .158) (Table 5 and Fig 2). These traits also significantly influenced negative academic emotion, with neuroticism (β = .214), self-critical perfectionism (β = .113), and impulsivity (β = .083). Needs frustration directly affected negative academic emotion (β = .284), performance expectations (β = .331), and ChatGPT dependency (β = .136). Performance expectations were positively related to ChatGPT dependency (β = .242), while negative academic emotion did not have a direct effect. The model explained 14.6% of the variance in needs frustration, 22.7% in negative academic emotions, 32.5% in performance expectations, and 12.3% in ChatGPT dependency.

**Table 5:** Structural path analysis.

Path	*B*	β	*SE*	*t*	*p*
NEU → NF	.174	.169	.035	4.997	< .001
IM → NF	.191	.158	.04	4.753	< .001
SCP →NF	.252	.224	.04	6.308	< .001
NF → NAE	.276	.284	.033	8.397	< .001
NEU → NAE	.214	.214	.033	6.446	< .001
IM → NAE	.097	.083	.038	2.572	.01
SCP → NAE	.123	.113	.038	3.269	.001
NAE → PE	.384	.353	.038	10.231	< .001
NF → PE	.349	.331	.036	9.656	< .001
PE → CD	.210	.242	.039	5.381	< .001
NF → CD	.125	.136	.037	3.365	< .001
NAE → CD	.038	.04	.039	.976	.329

*Note*: *B* = unstandardized estimates, β = standardized estimates, NEU = neuroticism, SCP = self-critical perfectionism, IM = impulsivity, NF = needs frustration, NAE = negative academic emotion, PE = performance expectation, CD = ChatGPT dependency.

#### Mediation and serial mediation analysis

Table 6 presents the results of the indirect analyses of each personality trait. The indirect effects of neuroticism (β = .023, 95% CI [.009-.047]), self-critical perfectionism (β = .03, 95% CI [.012-.056]), and impulsivity (β = .021, 95% CI [.008-.043]) on ChatGPT dependency through needs frustration were significant. Serial mediation effects were also found significant through needs frustration and performance expectations for neuroticism (β = .013, 95% CI [.007-.024]), self-critical perfectionism (β = .018, 95% CI [.01-.03]), and impulsivity (β = .013, 95% CI [.002-.016]). Finally, the serial mediation involving needs frustration, negative academic emotion, and performance expectations significantly mediated the influence of neuroticism (β = .004, 95% CI [.002-.008]), self-critical perfectionism (β = .005, 95% CI [.003-.01]), and impulsivity (β = .004, 95% CI [.002-.008]) and ChatGPT dependency.

**Table 6:** Indirect path analysis.

Indirect Path	β	Boot *SE*	Lower CI	Higher CI	*p*
**Neuroticism**					
NEU → NF → CD	.023	.009	.009	.047	.001
NEU → NF → PE → CD	.013	.004	.007	.024	< .001
NEU → NAE → PE → CD	.018	.005	.01	.032	< .001
NEU →NF →NAE → PE → CD	.004	.001	.002	.008	< .001
Total indirect effect	.059	.013	.038	.089	< .001
**Self-Critical Perfectionism**					
SCP → NF → CD	.03	.011	.012	.056	.002
SCP → NF → PE → CD	.018	.005	.01	.03	< .001
SCP → NAE → PE → CD	.01	.004	.004	.019	< .001
SCP → NF → NAE → PE → CD	.005	.002	.003	.01	< .001
Total indirect effect	.063	.013	.041	.093	< .001
**Impulsivity**					
IM → NF → CD	.021	.009	.008	.043	.001
IM → NF → PE → CD	.013	.004	.006	.023	< .001
IM → NAE → PE → CD	.007	.003	.002	.016	.008
IM → NF → NAE → PE → CD	.004	.001	.002	.008	< .001
Total indirect effect	.045	.011	.026	.07	< .001

*Note*: NEU = neuroticism, SCP = self-critical perfectionism, IM = impulsivity, NF = needs frustration, NAE = negative academic emotion, PE = performance expectation, CD = ChatGPT dependency. Confidence intervals were calculated at a 95% confidence level.

#### Exploratory analysis

During the review process, we were requested to conducting an additional exploratory analysis to examine the influence of demographic variables on needs frustration and ChatGPT dependency, aiming to explore nuanced differences in participants’ demographic backgrounds. The analysis revealed that only gender and degree level had a statistically significant impact on needs frustration, while other demographic factors, such as academic discipline and frequency of ChatGPT usage, showed no significant effects on either needs frustration or ChatGPT dependency. Gender demonstrated a significant influence, with females reporting higher levels of needs frustration compared to males (β = 0.095, 95% CI [0.067, 0.290], *p* = 0.002). In terms of degree level, there was a negative relationship between academic progression and needs frustration (β = -0.098, 95% CI [-0.220, -0.050], *p* = 0.001), indicating that students at higher academic levels (e.g., Ph.D.) experienced lower needs frustration than those at lower levels (e.g., undergraduate). Other variables, including study discipline and ChatGPT use frequency, did not significantly predict needs frustration or ChatGPT dependency.

## Discussion

This study demonstrates that neuroticism, self-critical perfectionism, and impulsivity are key personality traits contributing to dependency on ChatGPT among university students, primarily through their impact on needs frustration, cognitive biases, and performance expectations. These findings emphasize the importance of addressing both emotional and cognitive factors to mitigate AI dependency in educational contexts. By applying the Interaction of Person-Affect-Cognition-Execution (I-PACE) model and Basic Psychological Needs (BPN) theory, this study advances the understanding of how unmet psychological needs drive maladaptive dependencies, aligning with prior research linking needs frustration to technology overuse [51, 52].

### Personality and AI dependency

Our findings show that neuroticism emerged as the strongest predictor of negative academic emotions, suggesting that individuals high in neuroticism are particularly vulnerable to academic stressors. The AI tool’s ability to provide instant feedback and solutions offers a temporary sense of control and competence, making it especially appealing to those with high neuroticism. This finding aligns with previous research linking neuroticism to digital dependency, where neurotic individuals use tools such as social media to manage emotional distress [53, 54]. However, our study extends this understanding by highlighting the unique academic context of ChatGPT use. Unlike social media, which primarily provides emotional gratification, ChatGPT directly impacts students’ perceived competence and control over academic tasks. This creates a specific cognitive bias where neurotic individuals may feel that their academic success depends on the tool, potentially leading to overreliance as a crutch to avoid confronting academic insecurities and emotional distress.

Self-critical perfectionism also plays a significant role, characterized by fear of underperforming and tendencies toward procrastination [55], Such individuals may find ChatGPT particularly appealing because it offers immediate feedback and reassurance, helping them manage procrastination and alleviate the fear of failure. However, this reliance can prevent the development of resilience and problem-solving skills, reinforcing feelings of inadequacy and fostering a cycle of dependency [56].

Impulsivity, though a less consistent predictor, was still significantly associated with needs frustration and negative emotions. Impulsive individuals, who often act without forethought, may turn to AI tools as a quick fix for academic challenges, aligning with previous findings that link impulsivity to maladaptive technology use [57]. This reliance on ChatGPT is further reinforced by avoidance behavior characteristics since they prefer easier routes to complete tasks and avoid the efforts associated with thorough planning and critical thinking. This avoidance behavior intensifies under high academic pressure, making ChatGPT an attractive option because of its ability to deliver straightforward answers quickly.

### Cognitive factors as key drivers

A key finding is that negative academic emotions alone does not mediate the relationship between personality traits and ChatGPT dependency. Instead, the combined influence of negative emotions and performance expectations is crucial in fostering dependency. This aligns with cognitive-behavioral models, suggesting that negative self-appraisal, coupled with cognitive biases, can lead to maladaptive technology use [58]. Previous research by Casale [59] and Brand [17] has shown that positive metacognitions about technology use mediate the link between emotional dysregulation and technology reliance, supporting our findings. Thus, students experiencing frustration may hold negative self-appraisals and doubts about their self-efficacy, leading them to develop a strong belief in ChatGPT as a coping mechanism. Furthermore, the finding that degree level significantly predicts needs frustration, but not ChatGPT dependency directly, suggests that students in more advanced academic programs tend to develop greater cognitive resilience and problem-solving skills. This resilience may buffer them from frustration when facing academic challenges, thus reducing their likelihood of becoming overly dependent on tools like ChatGPT. Gender differences further contribute to this dynamic. Female students reported higher levels of needs frustration, indicating that they are more susceptible to emotional distress in academic settings. However, gender did not directly influence ChatGPT dependency. This suggests that while women may experience greater academic frustration, other cognitive factors—such as cognitive resilience and self-efficacy beliefs—likely play a more critical role in determining technology dependency.

Technologically, the perceived usefulness and ease of use of ChatGPT are well-documented antecedents of technology acceptance in educational settings [60]. The interactive nature of ChatGPT, providing quick answers with minimal input, makes it easily accessible to students. Although this study did not directly measure perceptions of ChatGPT, the high frequency of use reported (42.2% daily, 40.6% very often) suggests its perceived value. From a broader perspective, research shows that people often have unrealistic expectations of a technology’s ability to bring about positive change, akin to a placebo effect observed in digital interventions [61, 62]. In the context of ChatGPT, students may develop an illusion of efficacy, fostering dependency.

### Implications

This study contributes to personality psychology by demonstrating that specific traits—neuroticism, self-critical perfectionism, and impulsivity—affect dependency on Generative AI tools like ChatGPT. By applying the I-PACE model, the study validates the model’s emphasis on the interaction between individual differences and affective-cognitive processes in understanding technology dependency. Additionally, the integration of BPN theory highlights how needs frustration mediates the relationship between personality traits and AI use, providing a nuanced understanding of the psychological mechanisms driving dependency. These findings suggest a refinement of current models to account for the specific role of cognitive expectations in fostering AI dependency.

Practically, these findings suggest the need for tailored interventions that address both the emotional and cognitive aspects of AI dependency. For individuals prone to neuroticism, self-critical perfectionism, and impulsivity, educators could implement strategies that focus on emotional regulation skills, such as mindfulness-based stress reduction, and cognitive restructuring techniques to challenge maladaptive thought patterns. Technology developers could design AI tools with built-in features that promote self-regulation and provide feedback on healthy usage patterns. Additionally, psycho-educational programs that help students understand the realistic capabilities and limitations of AI tools could reduce overreliance and dependency. Moreover, cognitive-behavioral strategies, such as cognitive restructuring, could also help challenge negative self-appraisals and enhance coping mechanisms. Interventions targeting performance expectations could help students develop more balanced and realistic appraisals of their abilities, reducing the perceived need to rely on AI tools as a crutch. Such approaches may reduce needs frustration, manage performance expectations, and promote a healthier, more effective use of AI tools in educational contexts.

### Limitations and future directions

This study’s cross-sectional design limits the ability to draw causal inferences, meaning that the directionality of the relationships observed cannot be definitively established. Future research employing longitudinal approaches could better capture the dynamic evolution of AI dependency over time. Additionally, the study’s focus on Chinese university students may limit the generalizability of the findings to other cultural contexts. Variations in educational practices, cultural attitudes toward technology, and personality trait expressions suggest that cross-cultural replication studies are needed to assess the robustness of these findings. Cultural variables, such as the norms and values of different countries and educational institutions, may moderate the relationship between personality traits and AI dependency. Exploring the interaction between personality traits and cultural factors in future studies could offer more nuanced insights into how these relationships manifest in various cultural settings. Moreover, expanding the range of personality traits examined could provide a more comprehensive understanding of how different personality dimensions interact with technology use behaviors. Additionally, researchers could investigate the specific technical features of AI tools that contribute to dependency, as understanding both personal characteristics and technological attributes may deepen insights into the factors driving dependency. By combining these perspectives, future research could provide a more holistic view of how individual, technological and cultural factors interact in shaping AI dependency.

## Conclusion

This study highlights the significant influence of personality traits, including neuroticism, self-critical perfectionism, and impulsivity on dependency of Generative AI tools like ChatGPT, primarily through their impacts on needs frustration, negative emotional, and performance expectations. By uncovering these psychological drivers, we provide a basis for developing interventions aimed at fostering healthier interactions between students and AI technologies, while also supporting their academic well-being. Future research should expand on these findings by examining these relationships over time and within diverse cultural contexts to further understand the broader implications of AI dependency.

## Supporting information

S1 Table Zero-order correlations. (DOCX)

S2 Table Factor loadings, reliability and validity. (DOCX)

S3 Table Bootstrapping bias correction for the structural model. (DOCX)
