# Supplementary material for: How Do Personal Attributes Shape AI Dependency in Chinese Higher Education Context? Insights from Needs Frustration Perspective
Source: PLoS One. 2024 Nov 1;19(11):e0313314. doi: 10.1371/journal.pone.0313314 (PMC11530054; doi:10.1371/journal.pone.0313314)
Supplement: S1 Table — (DOCX) [file pone.0313314.s001.docx]

**S1 Table. Zero-order Correlations**

| **Constructs** | ***M*** | ***SD*** | **1** | **2** | **3** | **4** | **5** | **6** | **7** | **8** | **9** | **10** | **11** | **12** | **13** | **14** | **15** | **16** |
| --- | --- | --- | --- | --- | --- | --- | --- | --- | --- | --- | --- | --- | --- | --- | --- | --- | --- | --- |
| **1** Neuroticism | 4.552 | .631 | [.94] |  |  |  |  |  |  |  |  |  |  |  |  |  |  |  |
| **2** Self-Critical Perfectionism | 3.868 | .914 | .177^**^ | [.876] |  |  |  |  |  |  |  |  |  |  |  |  |  |  |
| **3** Non-Planning Impulsivity | 3.77 | 1.08 | .197^**^ | .203^**^ | [.906] |  |  |  |  |  |  |  |  |  |  |  |  |  |
| **4** Motor Impulsivity | 3.792 | 1.09 | .186^**^ | .184^**^ | .913^**^ | [.908] |  |  |  |  |  |  |  |  |  |  |  |  |
| **5** Attentional Impulsivity | 3.786 | 1.076 | .201^**^ | .21^**^ | .909^**^ | .902^**^ | [.905] |  |  |  |  |  |  |  |  |  |  |  |
| **6** Impulsivity (2nd order) | 3.782 | 1.048 | .201^**^ | .205^**^ | .971^**^ | .969^**^ | .967^**^ | [.967] |  |  |  |  |  |  |  |  |  |  |
| **7** Autonomy Frustration | 3.701 | .1.014 | .198^**^ | .24^**^ | .203^**^ | .188^**^ | .204^**^ | .205^**^ | [.846] |  |  |  |  |  |  |  |  |  |
| **8** Relatedness Frustration | 3.716 | 1.035 | .218^**^ | .26^**^ | .214^**^ | .192^**^ | .21^**^ | .212^**^ | .843^**^ | [.847] |  |  |  |  |  |  |  |  |
| **9** Competence Frustration | 3.710 | 1.004 | .221^**^ | .244^**^ | .231^**^ | .219^**^ | .237^**^ | .237^**^ | .839^**^ | .852^**^ | [.838] |  |  |  |  |  |  |  |
| **10** Needs Frustration (2nd order) | 3.71 | .964 | .224^**^ | .262^**^ | .228^**^ | .211^**^ | .229^**^ | .23^**^ | .944^**^ | .949^**^ | .947^**^ | [.942] |  |  |  |  |  |  |
| **11** Irritation | 3.752 | .983 | .28^**^ | .213^**^ | .192^**^ | .174^**^ | .196^**^ | .193^**^ | .317^**^ | .322^**^ | .333^**^ | .342^**^ | [.833] |  |  |  |  |  |
| **12** Anxiety | 3.80 | .99 | .294^**^ | .224^**^ | .207^**^ | .198^**^ | .211^**^ | .212^**^ | .34^**^ | .331^**^ | .327^**^ | .352^**^ | .831^**^ | [.84] |  |  |  |  |
| **13** Hopelessness | 3.771 | 1.01 | .272^**^ | .218^**^ | .184^**^ | .179^**^ | .198^**^ | .193^**^ | .327^**^ | .328^**^ | .332^**^ | .347^**^ | .843^**^ | .841^**^ | [.843] |  |  |  |
| **14** Negative Academic Emotion (2nd order) | 3.773 | .939 | .298^**^ | .231^**^ | .206^**^ | .194^**^ | .213^**^ | .211^**^ | .347^**^ | .346^**^ | .35^**^ | .367^**^ | .943^**^ | .943^**^ | .948^**^ | [.94] |  |  |
| **15** Performance Expectation | 3.582 | 1.048 | .287^**^ | .245^**^ | .229^**^ | .227^**^ | .246^**^ | .242^**^ | .413^**^ | .401^**^ | .401^**^ | .428^**^ | .423^**^ | .428^**^ | .394^**^ | .439^**^ | [.895] |  |
| **16** ChatGPT Dependency | 3.921 | .858 | .252^**^ | .149^**^ | .176^**^ | .16^**^ | .17^**^ | .174^**^ | .207^**^ | .228^**^ | .23^**^ | .234^**^ | .171^**^ | .185^**^ | .166^**^ | .184^**^ | .285^**^ | [.863] |

*Note:* ^**^*p* < .01; MacDonald’s ω coefficient along the diagonal within the bracket; *Mean* and *SD* for Non-Planning Impulsivity, Motor Impulsivity, Attentional Impulsivity and Impulsivity (2nd order) are rescaled to a 5-point scale.
