# Supplementary material for: How Do Personal Attributes Shape AI Dependency in Chinese Higher Education Context? Insights from Needs Frustration Perspective
Source: PLoS One. 2024 Nov 1;19(11):e0313314. doi: 10.1371/journal.pone.0313314 (PMC11530054; doi:10.1371/journal.pone.0313314)
Supplement: S2 Table — (DOCX) [file pone.0313314.s002.docx]

**S2 Table. Factor Loadings, Reliability and Validity**

| **Construct** | **Items** | **Factor Loading** | **CR** | **AVE** |
| --- | --- | --- | --- | --- |
| **Neuroticism** | NEU1 | .744 | .94 | .567 |
|  | NEU2 | .775 |  |  |
|  | NEU3 | .790 |  |  |
|  | NEU4 | .745 |  |  |
|  | NEU5 | .769 |  |  |
|  | NEU6 | .764 |  |  |
|  | NEU7 | .748 |  |  |
|  | NEU8 | .729 |  |  |
|  | NEU9 | .734 |  |  |
|  | NEU10 | .738 |  |  |
|  | NEU11 | .749 |  |  |
|  | NEU12 | .752 |  |  |
| **Self-Critical Perfectionism** | SCP1 | .722 | .877 | .542 |
|  | SCP2 | .738 |  |  |
|  | SCP3 | .715 |  |  |
|  | SCP4 | .739 |  |  |
|  | SCP5 | .780 |  |  |
|  | SCP6 | .721 |  |  |
| **Impulsivity** | ***NP*** | .959 | .967 | .908 |
|  | ***MT*** | .951 |  |  |
|  | ***AT*** | .948 |  |  |
| ***NP*** | IM1 | .818 | .906 | .658 |
|  | IM2 | .810 |  |  |
|  | IM3 | .818 |  |  |
|  | IM4 | .814 |  |  |
|  | IM5 | .795 |  |  |
| ***MT*** | IM6 | .807 | .908 | .664 |
|  | IM7 | .815 |  |  |
|  | IM8 | .838 |  |  |
|  | IM9 | .803 |  |  |
|  | IM10 | .811 |  |  |
| ***AT*** | IM11 | .807 | .905 | .655 |
|  | IM12 | .815 |  |  |
|  | IM13 | .808 |  |  |
|  | IM14 | .802 |  |  |
|  | IM15 | .815 |  |  |
| **Needs Frustration** | ***AF*** | .911 | .942 | .844 |
|  | ***RF*** | .924 |  |  |
|  | ***CF*** | .921 |  |  |
| ***AF*** | AF1 | .745 | .846 | .587 |
|  | AF2 | .784 |  |  |
|  | AF3 | .748 |  |  |
|  | AF4 | .764 |  |  |
| ***RF*** | RF1 | .76 | .848 | .582 |
|  | RF2 | .769 |  |  |
|  | RF3 | .752 |  |  |
|  | RF4 | .77 |  |  |
| ***CF*** | CF1 | .746 | .838 | .563 |
|  | CF2 | .736 |  |  |
|  | CF3 | .765 |  |  |
|  | CF4 | .754 |  |  |
| **Negative Academic Emotion** | ***IR*** | .913 | .939 | .838 |
|  | ***AX*** | .913 |  |  |
|  | ***HO*** | .920 |  |  |
| ***IR*** | IRRI1 | .749 | .832 | .554 |
|  | IRRI2 | .758 |  |  |
|  | IRRI3 | .721 |  |  |
|  | IRRI4 | .749 |  |  |
| ***AX*** | ANXI1 | .762 | .842 | .571 |
|  | ANXI2 | .749 |  |  |
|  | ANXI3 | .762 |  |  |
|  | ANXI4 | .749 |  |  |
| ***HO*** | HOPE1 | .757 | .843 | .572 |
|  | HOPE2 | .76 |  |  |
|  | HOPE3 | .758 |  |  |
|  | HOPE4 | .751 |  |  |
| **Performance Expectation** | PE1 | .767 | .895 | .586 |
|  | PE2 | .748 |  |  |
|  | PE3 | .773 |  |  |
|  | PE4 | .763 |  |  |
|  | PE5 | .779 |  |  |
|  | PE6 | .762 |  |  |
| **ChatGPT Dependency** | CD1 | .75 | .863 | .512 |
|  | CD2 | .704 |  |  |
|  | CD3 | .707 |  |  |
|  | CD4 | .702 |  |  |
|  | CD5 | .702 |  |  |
|  | CD6 | .727 |  |  |

*Note:* NP = Non-Planning Impulsivity, MT = Motor Impulsivity, AT = Attentional Impulsivity, AF = Autonomy Frustration, RF = Relatedness Frustration, CF = Competence Frustration, IR = Irritation, AX = Anxiety, HO = Hopelessness.
