# Supplementary material for: How Do Personal Attributes Shape AI Dependency in Chinese Higher Education Context? Insights from Needs Frustration Perspective
Source: PLoS One. 2024 Nov 1;19(11):e0313314. doi: 10.1371/journal.pone.0313314 (PMC11530054; doi:10.1371/journal.pone.0313314)
Supplement: S3 Table — (DOCX) [file pone.0313314.s003.docx]

**S3 Table. Bootstrapping Bias Correction for Structural Model**

| **Parameter** | ***SE*** | ***SE-SE*** | ***Bias*** | ***SE-Bias*** |
| --- | --- | --- | --- | --- |
| NEU 🡪 NF | .037 | .00 | .00 | .001 |
| IM 🡪 NF | .043 | .00 | .00 | .001 |
| SCP 🡪 NF | .044 | .00 | .001 | .001 |
| NF 🡪 NAE | .037 | .00 | .00 | .001 |
| NEU 🡪 NAE | .037 | .00 | .00 | .001 |
| IM 🡪 NAE | .041 | .00 | .00 | .001 |
| SCP 🡪 NAE | .039 | .00 | .001 | .001 |
| NAE 🡪 PE | .043 | .00 | .00 | .001 |
| NF 🡪 PE | .04 | .00 | .001 | .001 |
| PE 🡪 CD | .043 | .00 | .00 | .001 |
| NF 🡪 CD | .041 | .00 | .00 | .001 |
| NAE 🡪 CD | .04 | .00 | .00 | .001 |

*Note*. NEU = Neuroticism, SCP = Self-Critical Perfectionism, IM = Impulsivity, NF = Needs Frustration, NAE = Negative Academic Emotion, PE = Performance Expectation, CD = ChatGPT Dependency, SE-SE = Standard Error of Standard Error, SE-Bias = Standard Error Bias.
